# Male osteoporosis

**DOI:** 10.20945/2359-3997000000563

**Published:** 2022-11-11

**Authors:** Leonardo Bandeira, Barbara C. Silva, John P. Bilezikian

**Affiliations:** 1 Universidade Federal de São Paulo São Paulo SP Brasil Universidade Federal de São Paulo, São Paulo, SP, Brasil; 2 Grupo Fleury A+ Medicina Diagnóstica Pernambuco PE Brasil A+ Medicina Diagnóstica, Grupo Fleury, Pernambuco, PE, Brasil; 3 Centro Universitário de Belo Horizonte Departamento de Medicina Belo Horizonte MG Brasil Departamento de Medicina, Centro Universitário de Belo Horizonte (UNI-BH), Belo Horizonte, MG, Brasil; 4 Hospital Felício Rocho Unidade de Endocrinologia Belo Horizonte MG Brasil Unidade de Endocrinologia, Hospital Felício Rocho, Belo Horizonte, MG, Brasil; 5 Hospital Santa Casa Unidade de Endocrinologia Belo Horizonte MG Brasil Unidade de Endocrinologia, Hospital Santa Casa, Belo Horizonte, MG, Brasil; 6 Columbia University College of Physicians & Surgeons Metabolic Bone Diseases Unit New York NY USA Division of Endocrinology, Metabolic Bone Diseases Unit, College of Physicians & Surgeons, Columbia University, New York, NY, USA

**Keywords:** Osteoporosis, male, hypogonadism, testosterone

## Abstract

Osteoporosis, a disease classically attributed to postmenopausal women, is underappreciated, underdiagnosed, and undertreated in men. However, it is not uncommon for osteoporotic fractures to occur in men. About 40% of fractures occur in men with an incidence that has increased over the years. After a first fracture, the risk of a subsequent episode, as well as the risk of death, is higher in the male than in the female population. Despite these facts, only 10% of men with osteoporosis receive adequate treatment. Up to half of the cases of male osteoporosis have a secondary cause, the most common being hypogonadism, excessive alcohol consumption, and chronic use of glucocorticoids. The International Society for Clinical Densitometry (ISCD) recommends using the female database for the diagnosis of osteoporosis by DXA (T-score ≤ −2.5 in men over 50 years old). In addition, osteoporosis can also be diagnosed independently of the BMD if a fragility fracture is present, or if there is a high risk of fractures by FRAX. Treatment is similar to postmenopausal osteoporosis, because the data regarding changes in bone density track closely to those in women. Data concerning fracture risk reduction are not as certain because the clinical trials have included fewer subjects for shorter period of time. In men with symptomatic hypogonadism, testosterone replacement, if indicated, can improve BMD.

## INTRODUCTION

Osteoporosis is a disease characterized by reduced bone mass and deterioration of skeletal microarchitecture, resulting in increased bone fragility and risk of fractures. It is the most common metabolic bone disease and its main outcome, osteoporotic fractures, significantly increase morbidity and mortality. Traditionally a disease of women, osteoporosis is underappreciated, underdiagnosed, and undertreated in men. Although more common in women, the disease occurs with considerable frequency in the male population, with a prevalence of about 12% worldwide and reaching more than 20% in some regions of the world (1–3). Furthermore, the morbidity and mortality due to fractures are significantly higher in men than in women, perhaps because they occur 5 to 10 years later in men, and thus, are accompanied by more comorbidities (4,5). This narrative review provides an update on several aspects of male osteoporosis.

## EPIDEMIOLOGfY

The prevalence of osteoporosis and fractures has increased in the last decades more sharply in the male than in the female population. Between the early 1990s and mid-2000s, the prevalence of osteoporosis and osteopenia doubled in men over 50 years old in the US, reaching 4% and 38%, respectively (6). The chance of a man suffering an osteoporotic fracture during his lifetime is greater than that of developing prostate cancer. In 2000, 40% of the 9 million fractures that occurred in the world were in men (7). In addition, a 310% increase in hip fractures is expected between 1990 and 2050 in this population.

A cohort study with 234 men over 60 years old showed a 32% prevalence of morphometric vertebral fractures (8). In another study with 725 men over 40 years old from the 5 Brazilian regions, the prevalence of fragility fractures was 12.8% (9). The increase of osteoporotic fractures in this population is highly relevant, since after a first fracture, the risk of a subsequent fracture, as well as the risk of death, is higher in the male than in the female population (4). Still, less than 20% of men with osteoporosis receive treatment, even considering those with previous fractures (10,11).

## PATHOPHYSIOLOGY

The lower prevalence of male osteoporosis, when compared to female, can be explained by several factors. Peak bone mineral density (BMD) is 8% to 10% higher in men than women. Thus, men are generally endowed with greater peak bone mass before they begin to lose bone mass. In addition, bone size is generally greater in men, which confers a mechanical advantage, as stressors are distributed over a wider cross-sectional area. Increased bone size can be accounted for by the action of androgens influencing periosteal bone formation in men. With age, bone external diameter increases, further in men than in women, perhaps in compensation for endocortical thinning and increased cortical porosity. This significant compensatory increase in bone diameter, due to the action of androgens on periosteal apposition is likely to be a key differentiating point between men and women as they age. Another differentiating point is related to trabecular microstructure. Men have more bone trabeculae than women, and with aging, the pattern of trabecular loss is different. In men, aging is associated with trabecular thinning, but not loss, while in women, aging is more likely to be associated with trabecular perforations and loss of trabecular connectivity. These points are characteristic of accelerated bone resorption state associated with the menopause (12–15).

Unlike women, where menopause is associated with a rather abrupt drop in estrogen levels, androgen levels decline much more slowly and gradually in males. An acceleration of this process can occur in men, but usually at a more advanced age (after 70 years old). In special situations in which a hypogonadal state ensues either due to illness or to medication, a sudden drop in testosterone levels can lead to an increase in bone resorption, rapid bone loss, and a greater risk of fractures, similar to what is seen in postmenopausal women who are not treated with estrogen (16–20). Increases in sex hormone-binding globulin (SHBG) with a reduction in free testosterone levels are also associated with a decrease in BMD and an increased risk of fractures (21,22).

Estrogen levels also seem to play a fundamental role in the male skeleton. Its sufficiency is important for reaching peak bone mass and its deficiency is directly associated with the increased remodeling and bone loss in men (23,24). Prospective multicenter studies have shown that reduced serum estradiol is an independent risk factor for fractures in men (25,26). Those patients who have the combination of low levels of estradiol and free testosterone are at the greatest risk for bone loss and fractures (21,22,25,26). Reduced dehydroepiandrosterone (DHEA) levels have also been associated with lower BMD, muscle weakness and risk of falls, and a higher risk of fractures in older men (27).

In addition to mechanical and hormonal aspects, there are those specifically associated with aging, since in men bone loss occurs at an older age compared with women. Inflammatory cytokines in elderly people can lead to an increase in osteoclast activity and inhibition of osteoblasts (28). Moreover, the elderly have a higher prevalence of vitamin D deficiency due to several factors (dietary restriction, less sun exposure, and lower cutaneous conversion capacity of 7-dehydrocholesterol into cholecalciferol) as well as declining muscle function leading to more falls (29–31).

## ETIOLOGY

Secondary causes of osteoporosis are more common in men than in postmenopausal women. Still, a specific cause can be found in only 40% to 50% of men with osteoporosis. The main causes of osteoporosis in the male population are hypogonadism, excessive alcohol consumption, and chronic use of glucocorticoids. Other causes are described in Table 1. Secondary causes should be investigated in all men with osteoporosis. In addition to a careful history and physical examination, laboratory evaluation is recommended. Initial laboratory testing should include complete blood count, serum creatinine, calcium, phosphorus, alkaline phosphatase, liver function tests, testosterone, 25-OH-vitamin D, TSH, in addition to analysis of 24-hour urine calcium, sodium and creatinine excretion. This workup identifies approximately 90% of occult disorders (32). Further evaluation is indicated when there is suspicion for specific conditions. When a specific cause is not identified, osteoporosis is idiopathic. In the elderly, it can be referred to as age-related (10). Idiopathic osteoporosis is found mainly in middle-aged men. Most of these patients appear to have a low turnover state, with reduced bone formation (33).

**Table 1:** Etiology of male osteoporosis

Primary	Secondary
Idiopathic osteoporosis	Medications: Glucocorticoid GnRH analogs Aromatase inhibitors Anticonvulsants Excessive dose of levothyroxine Chemotherapy
Age-related osteoporosis	Endocrine disorders: Hyperparathyroidism Hyperthyroidism Hypogonadism Hypopituitarism Acromegaly Cushing's syndrome Diabetes mellitus
	Gastrointestinal disorders: Celiac disease Inflammatory bowel disease Post-gastrectomy Other malabsorptive syndromes Primary biliary cirrhosis
	Rheumatological diseases: Rheumatoid arthritis Ankylosing spondylitis Systemic lupus erythematosus
	Hematological diseases: Multiple myeloma Leukemia, lymphoma Mastocytosis Hemophilia Thalassemia
	Genetic diseases: Idiopathic hypercalciuria Osteogenesis imperfecta Hypophosphatasia Homocystinuria Klinefelter syndrome Hemochromatosis
	Miscellaneous: Vitamin D deficiency Chronic obstructive pulmonary disease Post-transplant Malignant neoplasm Heart failure Chronic kidney disease Abusive use of alcohol

## DIAGNOSIS

As in females, the gold standard for diagnosing osteoporosis in males is the measurement of BMD by dual-energy X-ray absorptiometry (DXA). In men over 50 years old, the disease is defined by a T-score ≤ −2.5. The recommendation of the International Society of Clinical Densitometry (ISCD) is to use the female database as a reference for the T-score in men (34). This recommendation may underestimate the prevalence of osteoporosis in this population since peak bone mass is higher in men than in women. On the other hand, for a given BMD in g/cm^2^, fracture risk is virtually identical between men and women, validating this recommendation in clinical practice (35). Screening for osteoporosis in men by DXA is recommended for all over 70 years old, or earlier when the risk of fractures is substantial higher than the normal population such as hypogonadism, chronic use of glucocorticoids, alcohol abuse, and previous fracture (36).

When osteoporosis is diagnosed in men, the standard of care is to treat with a drug approved for men. In other situations, such as in men with osteopenia whose fracture risk is high as determined by FRAX (Fracture Risk Assessment Tool), treatment also is recommended. FRAX incorporates well-established independent risk factors to calculate the absolute risk of fractures in 10 years (37). A risk percentage is given for any major osteoporotic fracture and separately for a hip fracture. The calibration of the algorithm used in FRAX and its interpretation vary among countries, as they depend on local epidemiological data and, in some countries, the cost-effectiveness of the treatment (36).

The presence of a fragility fracture of the femur or vertebra regardless of BMD, or the pelvis, humerus, or forearm in the presence of osteopenia, also allows for the diagnosis of osteoporosis and pharmacological therapy is recommended (36,38).

Most vertebral fractures are asymptomatic. Imaging modalities can readily detect vertebral fractures (vertebral fracture assessment or spine X-ray) in patients with osteopenia associated with at least one of the following: men over 80 years old, historical height loss greater than 4 cm or 1.5 inches, self-reported but not recorded prior vertebral fracture or glucocorticoid therapy equivalent to 5 mg or more of prednisone per day for ≥ 3 months (34).

Other imaging techniques that assess bone microarchitecture such as HRpQCT (high resolution quantitative peripheral computed tomography) and TBS (trabecular bone score), despite not used for the diagnosis of osteoporosis, may be useful in further assessment of bone quality. While HRpQCT is still a research instrument available only in medical centers with research programs in osteoporosis, TBS is a simple tool derived from the evaluation of the lumbar spine DXA image, calculated through specific software (39–41). TBS can be incorporated into FRAX to adjust fracture risk calculation, and therefore, to assist in the decision to treat.

## TREATMENT

### Non-pharmacological

Nonpharmacological measures such as physical activity, limited alcohol intake, and smoking cessation should be recommended for all. In addition, vitamin D supplementation to maintain serum levels of 25-OH-vitamin D greater than 30 ng/mL, and a daily intake of 1000 to 1200 mg of calcium through diet or supplements are important nonpharmacological measures (42–47). If any secondary cause of osteoporosis is identified, it should be treated (36).

### Bisphosphonates

By inhibiting osteoclasts, bisphosphonates reduce bone resorption. Alendronate, risedronate, and zoledronic acid are the main representatives of this class, all of which are approved by the regulatory agencies for the treatment of osteoporosis in men. The effects of bisphosphonates on BMD and bone turnover appear to be similar between men and women (48).

In men, alendronate therapy for 2 years led to an increase in BMD in the lumbar spine (+7.1%), femoral neck (+2.5%), and total body (+2.0%), in addition to a reduction in bone turnover markers and the risk of morphometric vertebral fractures (incidence 0.8 vs. 7.1%, p = 0.02 compared with placebo) (49).

The use of risedronate for 2 years also led to a significant increase in BMD in the lumbar spine (+6.5%), femoral neck (+3.2%), and total femur (+4.4%), with a reduction of > 60% in the risk of vertebral fractures. The drug was also effective in preventing non-vertebral fractures (50).

A large prospective, multicenter, randomized study assessed the efficacy of zoledronic acid versus placebo for 2 years in 1,199 men with osteoporosis (either primary or secondary to hypogonadism). At 24 months, the group treated with zoledronic acid showed a 67% reduction in the relative risk of morphometric vertebral fractures (p = 0.002 compared with placebo), in addition to increased BMD and suppression of bone markers (51).

A meta-analysis of 22 randomized control trials, including 4,868 participants, that evaluated the efficacy of osteoporosis treatment in adult men and reported fractures outcomes, demonstrated a significantly lower risk of vertebral (RR: 0.368, 95%CI: 0.252-0.537) and non-vertebral fractures (RR: 0.604, 95%CI: 0.404-0.904) in bisphosphonate users than in placebo-treated subjects (52). Another meta-analysis comparing bisphosphonates in men suggested that zoledronic acid would be the most effective in preventing vertebral fractures while risedronate would be the most effective in preventing non-vertebral fractures (53). However, the authors highlight that the eligible studies were inadequate to strongly support these results, and that more well-designed studies are needed to compare the anti-fracture efficacy of different bisphosphonates in male osteoporosis.

There is no long-term study of bisphosphonates in men. However, based on postmenopausal women's data, most experts suggest reassessment after 5 years of oral and 3 years of intravenous bisphosphonate therapy. If the patient continues to be at high risk for fractures, treatment can be extended for up to ten years for oral or six years for intravenous bisphosphonates. Although rare, in some patients, long-term treatment may increase the risk of complications, such as atypical femur fractures and osteonecrosis of the jaw (54).

### Denosumab

By binding to nuclear factor-kappa beta activating receptor-ligand (RANKL), denosumab prevents the interaction between RANKL, a powerful bone-resorbing cytokine, to its receptor RANK on osteoclasts. This results in a powerful inhibition of these cells and thus an antiresorptive effect (55).

In a randomized control trial, men receiving androgen deprivation therapy for prostate cancer who used denosumab had a significant increase in BMD at all sites compared with placebo (6.7% in the lumbar spine, 4.8% in the total hip, 3.9% in the femoral neck, and 5.5% in the distal radius after 24 months, p < 0.002 for all; n = 734 for each group). After 36 months, there was a 62% reduction in the risk of vertebral fractures (1.5 vs. 3.9%, p = 0.006 compared with placebo). Bone turnover markers decreased significantly in the denosumab as compared to the placebo group (56).

Unlike bisphosphonates, discontinuation of denosumab is followed by a rapid increase in bone turnover markers, bone loss and greater risk of multiple vertebral fractures, as observed in postmenopausal women (57,58). For this reason, it is recommended not to stop the medication or that, if discontinued, it should be switched to another potent antiosteoporosis agent (e.g., bisphosphonate). When denosumab is used for a long period, turnover suppression may be so intense that two doses of zoledronic acid at a 6-month interval is sometimes necessary to avoid a rebound in bone turnover, rapid bone loss and increased fracture risk following the discontinuation of denosumab (59).

### Teriparatide

Teriparatide (PTH 1-34) comprises the initial 34 aminoacids of the 84-aminoacid PTH molecule. Insight into the therapeutic potential of PTH came from the observation that PTH, when administered intermittently and in low doses, stimulates bone formation. Thus, it is an osteoanabolic agent (60–62).

In men with osteoporosis, teriparatide rapidly increases BMD, as demonstrated by Orwoll *et al*. in a randomized trial with 437 men with low bone density. At month 11, those treated with teriparatide (20 mcg by subcutaneous injection daily) experienced a 5.9% gain in BMD at the lumbar spine (p < 0.001 vs. placebo) and a 1.5% gain at the femoral neck (p = 0.02 vs. placebo). Heralding this increase, bone turnover markers rose within weeks of administration (63). In a follow-up study, 18 months after teriparatide discontinuation, even without antiresorptive therapy to follow in the majority, subjects who had been treated with active drug still had an 83% lower incidence of moderate or severe vertebral fractures than the placebo group (p < 0.01) (64). Based on other observations, however, when teriparatide is stopped, it should be followed by an antiresorptive medication (e.g., bisphosphonates) to prevent bone loss (65).

In men and women with glucocorticoid-induced osteoporosis, teriparatide led to greater BMD gains and fewer vertebral fractures than alendronate (1.7% vs. 7.7%, p = 0.007) (66).

### Abaloparatide

Abaloparatide is an analogue of PTHrP, designed to maximize its interaction with the configuration of the PTH/PTHrP receptor that favors anabolic activity (67). A recent phase 3, randomized, double-blinded, placebo-controlled trial was conducted for 1 year in 225 men, 45-84 years old (68). Lumbar spine BMD, the primary endpoint, increased by 8.5% in the abaloparatide group compared to 1.2% in the placebo group (p < 0.0001). Significant increases in BMD in comparison to placebo were also seen at the total hip and femoral neck sites. Bone turnover markers showed the typical major increase in the bone formation marker P1NP (procollagen type I N-terminal propeptide), followed more slowly and to a lesser extent in the bone resorption marker, CTX (carboxy-terminal collagen crosslinks).

### Safety of teriparatide and abaloparatide

The US Food and Drug Administration (FDA) has removed the boxed warning for both teriparatide and abaloparatide with regard to osteosarcoma and to duration of use. This recommendation is based upon abundant surveillance data for teriparatide that did not show any oncogenic signals in human subjects after over a decade of careful monitoring (69,70).

### Testosterone

Hypogonadism is a major etiology of osteoporosis in men. Testosterone deficiency is known to reduce BMD and increase the risk of fractures in men (18,22,25), which would justify its use in hypogonadal patients with osteoporosis. Testosterone replacement increases BMD, reduces bone turnover markers, and improves bone strength in men with hypogonadism (71–73). On the other hand, its use in the elderly with normal serum testosterone levels did not change BMD (74). Data regarding the effectiveness of testosterone treatment in fracture reduction are limited (75).

The Endocrine Society guidelines for male osteoporosis recommend testosterone replacement in hypogonadal patients (testosterone levels below 200 ng/mL in at least 2 measurements) who are symptomatic or who have known causes of hypogonadism (e.g. pituitary or testicular disorder), if there are no contraindications. However, testosterone is not the first-line therapy for hypogonadal men who are at high risk of fractures. Rather, an approved medication for osteoporosis is recommended and should be associated with testosterone therapy if there is a formal indication for testosterone replacement. Also, if there is a contraindication to an approved agent for osteoporosis in patients at high risk of fractures and testosterone levels below 200 ng/dL, testosterone therapy may be an alternative even with no standard indication for testosterone replacement (36).

In patients who are not at high risk for fractures, testosterone may alleviate symptoms of hypogonadism and improve BMD. There is no recommendation for testosterone use in eugonadal patients (36).

### Romosozumab

Romosozumab, an antisclerostin antibody, was developed to block the effects of sclerostin, a regulator of the *Wnt* bone formation signalling pathway. By blocking sclerostin, romosozumab was designed to be an osteoanabolic agent. Indeed, it is. However, another effect of romosozumab is as an antiresorptive agent. This mechanism of action is unique in that it is a dual action drug, namely increasing bone formation and inhibiting bone resorption. In men, romosozumab led to significantly greater BMD gains compared with placebo after 12 months (lumbar spine 12.1% vs. 1.2%; total hip 2.5% vs. −0.5%; femoral neck 2.2% vs. −0.2%, respectively; p < 0.001 for all) (76). In women, a larger clinical trial with fracture endpoints showed that the drug reduces fractures (77). In men, these data on fracture efficacy are not available. The medication was approved by the FDA for the treatment of postmenopausal osteoporosis in 2019 but has not yet been approved for male osteoporosis.

### Selection of initial therapy

A new fracture risk stratification was recently proposed in postmenopausal women with osteoporosis, that included a “very high risk” category. Women are considered at very high risk of fracture if they have very low BMD, very high fracture probability by FRAX, a history of multiple fractures, recent fractures, or falls. In these patients, an anabolic or more potent antiresorptive agents such as zoledronic acid or denosumab may be recommended as an initial osteoporosis treatment (78,79).

Although one could consider these recommendations to guide the therapeutic decisions in male osteoporosis, there is no strong data to support this new fracture risk stratification nor this therapeutical approach in men. Moreover, it is important to note that romosozumab and abaloparatide are not yet approved agents for treating osteoporosis in men.

### Monitoring therapy

BMD at the hip and spine should be assessed every 1-2 years to monitor response to treatment. If BMD is stable, less frequent DXA measurements are reasonable (36).

Bone turnover markers (BTM) are useful to monitor response to treatment in postmenopausal women (36). Although analyses of BTM in men are scarcer than in women, previously published data indicate a broad analogy in men with what has been observed for women. Trials of pharmacological treatment of osteoporosis in men have shown a decrease in BTMs following the use of antiresorptives (51,56,80,81), and an increase with the use of teriparatide (63,82). Thus, although there is no robust data to support the use of BTMs in men, these data indicate that BTMs may be used to monitor and improve treatment compliance during treatment of male osteoporosis. In women, it is recommended to measure BTMs at baseline, and 3-6 months after initiating osteoporosis treatment. For antiresorptive drugs, it is recommended to assess the bone resorption marker CTX, while the measurement of the bone formation marker P1NP is most appropriate for monitoring anabolic therapy (36).

In conclusion, this review summarizes the most recent data on epidemiology, pathophysiology, etiology, diagnosis, and treatment of male osteoporosis. Despite a frequent condition, male osteoporosis is still underappreciated by physicians, with less than 20% of men being treated, even considering those with previous fractures. With the increase in life expectancy, osteoporosis has become more prevalent in men and its main outcome, namely fracture, underscores a major health burden in this population, in addition to remarkable costs for health systems around the world. Thus, it is urgent to increase awareness of male osteoporosis, reducing undertreatment and improving care for this population.
